# Functional Morphological Differentiation Associated with Dung-Relocation Strategies in True Dung Beetles (Coleoptera: Scarabaeinae)

**DOI:** 10.3390/insects17090968

**Published:** 2026-09-20

**Authors:** Zhehao Tian, Jianxiong Su, Sheng Li, Yijie Tong, Xinpu Wang, Ming Bai

**Affiliations:** 1 School of Forestry and Grassland Science, Ningxia University, Yinchuan 750021, China; tianzh0354@163.com; 2 Hebei Key Laboratory of Animal Diversity, College of Life Science, Langfang Normal University, Langfang 065000, China; 3 Academy of Plateau Science and Sustainability, Qinghai Normal University, Xining 810016, China; 4 State Key Laboratory of Animal Biodiversity Conservation and Integrated Pest Management, Institute of Zoology, Chinese Academy of Sciences, Beijing 100101, China; 18145051662@163.com (J.S.); lisheng@ioz.ac.cn (S.L.); tongyijie@ioz.ac.cn (Y.T.); 5 School of Agricultural Science, Jiangxi Agricultural University, Nanchang 330045, China

**Keywords:** morpho-functional regularities, resource-use strategy, quantitative analysis, allometry

## Abstract

Understanding how ecological diversification translates into morphological differentiation is central to explaining the evolution of organismal form and function. True dung beetles (Coleoptera: Scarabaeidae: Scarabaeinae) exhibit diverse resource-use strategies, mainly including tunneling and rolling behaviors, which impose different functional demands on locomotion, excavation, and dispersal. However, it is unclear whether these ecological differences are consistently associated with differentiation in functional traits, particularly across different body-size classes. Here, we compared five dung beetle species representing large and small tunnelers, large and small rollers, and a mixed-strategy species using external structural traits, functional trait analyses, principal component analysis, and allometric modeling. Allometric analyses indicated that most functional traits exhibited similar scaling relationships among species after false-discovery-rate correction, suggesting that structural differentiation was not primarily driven by body-size differences. Functional morphological analyses revealed clear differentiation among species with contrasting resource-use strategies, with variation mainly associated with foreleg proportions, hindleg elongation, and wing morphology. Size-matched comparisons further showed that rollers consistently possessed relatively longer hindlegs and more elongated hind-tibiae than tunnelers across body-size classes, whereas foreleg proportions and protibial structures displayed more variable patterns. The mixed-strategy species exhibited a mosaic combination of traits rather than strictly structural dependencies. These findings demonstrate that dung-relocation strategies are associated with different coordinated functional trait differentiation, highlighting a potential trade-off between excavation-related and locomotion/dispersal-related specialization.

## Simple Summary

True dung beetles play important ecological roles by removing and burying animal dung, which contributes to nutrient cycling and ecosystem functioning. Different dung beetle species use different strategies to exploit dung resources, mainly by digging tunnels or rolling dung balls away from the original resource. These contrasting behaviors may require different body structures, but whether such differences are consistently reflected in morphology across body sizes remains unclear. In this study, we compared multiple morphological traits among five dung beetle species representing tunneling, rolling, and mixed strategies. We found that traits related to posterior legs showed the most consistent differences between rollers and tunnelers, whereas other structures showed more species-specific variation. Our results highlight the importance of functional morphology in understanding how ecological strategies are linked to organismal form.

## 1. Introduction

Understanding how ecological diversification manifests in structural differences is central to explaining the evolution of organismal form and function. Differences in resource use expose organisms to coordinate with morpho-functional peculiarities, which may promote the evolution of distinct structural adaptations. However, whether ecological differences are consistently associated with structural differentiation, and whether such associations remain stable across body-size scales, remains poorly understood. True dung beetles (Coleoptera: Scarabaeidae: Scarabaeinae) provide an excellent system for addressing these questions because they exhibit diverse resource-use strategies while relying primarily on animal dung as a food and reproductive resource.

Scarabaeinae currently comprise 6997 described species in 287 genera and 26 tribes worldwide [1]. Because dung resources are spatially patchy, ephemeral, and variable in quality, dung beetles have evolved diverse strategies for resource acquisition, relocation, and nesting. Through dung removal and burial, they contribute to nutrient cycling, soil modification, plant growth, secondary seed dispersal, and parasite regulation, and are therefore recognized as important ecosystem engineers [2]. Recent trait-based approaches further suggest that ecological functions emerge from interactions among multiple organismal traits, including morpho-functional peculiarities, behavior, and life-history characteristics [3]. Their ecological diversity and behavioral specialization provide an ideal framework for examining how resource-use differences are reflected in organismal form.

Dung-relocation strategies in Scarabaeinae are commonly classified into the major categories of tunneling (paracoprid) and rolling (telecoprid) behaviors [4,5,6], although some species exhibit other specialized strategies, such as endocoprid and kleptocoprid behaviors. Tunnelers excavate underground nests near the dung source and transport dung into these chambers, whereas rollers detach dung portions, form them into balls, and transport them away before burial. Although these categories are not absolute and some species combine elements of both strategies [7,8,9,10], tunneling and rolling represent contrasting modes of resource relocation that impose different functional demands during movement, manipulation, and nesting.

Previous studies have demonstrated associations between dung beetle structural and functional groups, nesting behavior, resource relocation, and ecological performance [10,11,12,13]. These findings indicate that variation in structural traits can provide a link between ecological strategy and functional performance. However, existing studies have often focused on individual traits or broad functional categories rather than integrated evaluations of multiple structural modules [3,14,15]. In addition, body size represents an important source of variation because many structural traits scale with overall body dimensions. Consequently, observed differences among species may reflect both ecological differentiation and allometric effects. Recent ecomorphological studies have emphasized the importance of accounting for body size when interpreting dung beetle structural variation [16], while scaling relationships may differ among anatomical structures [17]. Therefore, integrating allometric analyses with size-matched comparisons is necessary to determine whether morphological patterns associated with resource-use strategies are consistent across body-size classes.

In this study, we compared five dung beetle species representing contrasting tunneling, rolling, and mixed resource-use strategies across different body-size classes. Using standardized measurements of external structures, structural comparisons, multivariate analyses, allometric models, and predefined size-matched contrasts, we investigated whether ecological strategies are associated with consistent differentiation in functional structural traits. Specifically, we asked whether (1) contrasting resource-use strategies produce distinct structural trait combinations, (2) these differences persist after accounting for body-size effects, and (3) particular functional traits show consistent associations with tunneling and rolling behaviors. By integrating multiple structural dimensions across ecological strategies and body-size scales, this study provides insights into how resource-use diversification is reflected in the functional morphology of dung beetles.

## 2. Materials and Methods

### 2.1. Study Species and Specimen Sampling

**Figure 1:**
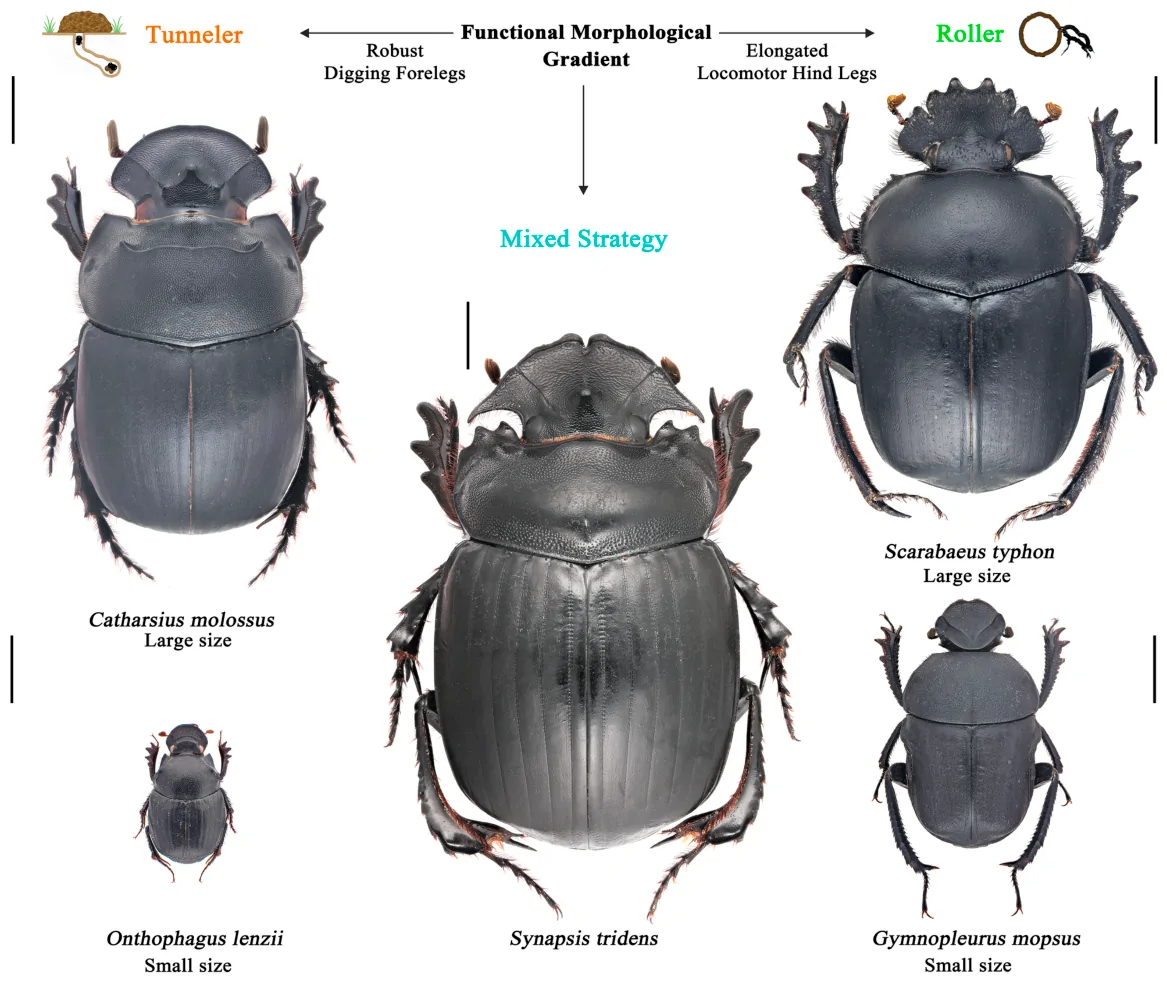
Structural differentiation among dung beetles with contrasting resource-use strategies. Scale bar = 5 mm.

Five dung beetle species representing contrasting resource-use strategies and body-size classes were selected for comparative morphological analysis (Figure 1). Resource-use strategies were classified according to dung-relocation and burial behavior. Tunnelers were defined as species that bury dung beneath or close to the dung source without transporting a discrete dung ball over an appreciable distance, whereas rollers detach a portion of dung, form it into a ball, and transport it away from the dung source before burial. *Catharsius molossus* and *Onthophagus lenzii* were classified as tunnelers, whereas *Scarabaeus typhon* and *Gymnopleurus mopsus* were classified as rollers [4]. *Synapsis tridens*, which exhibits short-distance dung rolling followed by burial, was operationally classified as having a mixed rolling–tunneling strategy.

To reduce the influence of large differences in overall body size, these four species were arranged into two predefined size-matched contrasts: a large-bodied contrast (*C. molossus* vs. *S. typhon*) and a small-bodied contrast (*O. lenzii* vs. *G. mopsus*). *S*. *tridens* was included to broaden the comparison of functional traits but was not included in the size-matched contrasts or assumed a priori to occupy an intermediate position in trait space.

Specimens were collected from five localities in China: *S. tridens* from Daxueshan Town, Yongde County, Lincang City, Yunnan Province; *O. lenzii* from Jinbaotun Town, Tongliao City, Inner Mongolia Autonomous Region; *S. typhon* from Jirigalangtu Town, Hanggin Banner, Ordos City, Inner Mongolia Autonomous Region; *G. mopsus* from Bayannur Town, Sonid Left Banner, Xilingol League, Inner Mongolia Autonomous Region; and *C. molossus* from Pidian Town, Zhengyang County, Zhumadian City, Henan Province.

Ten adult specimens were examined for each species, with five females and five males whenever possible. Specimens were preserved in 75% ethanol after collection.

### 2.2. Morphological Preparation, Imaging and Measurements

Before morphological examination and dissection, preserved specimens were relaxed in water heated to approximately 70 °C until the appendages and associated structures were sufficiently flexible for manipulation. Structural observations and dissections of legs, and hind-wings were conducted under an Olympus SZ61 stereomicroscope (Olympus, Tokyo, Japan). Left and right structures were designated according to their anatomical positions in ventral view of the head.

The legs, hind-wings and dorsal view of the specimen of *O. lenzii* were photographed using a Canon EOS 5DSR (Canon, Tokyo, Japan) equipped with a Mitutoyo M Plan Apo 5× objective (Mitutoyo, Kawasaki, Japan). The dorsal view of specimens of other species, as well as their legs and hind-wings, were photographed using the same camera equipped with a Canon EF 100 mm f/2.8 L Macro IS USM lens.

**Figure 2:**
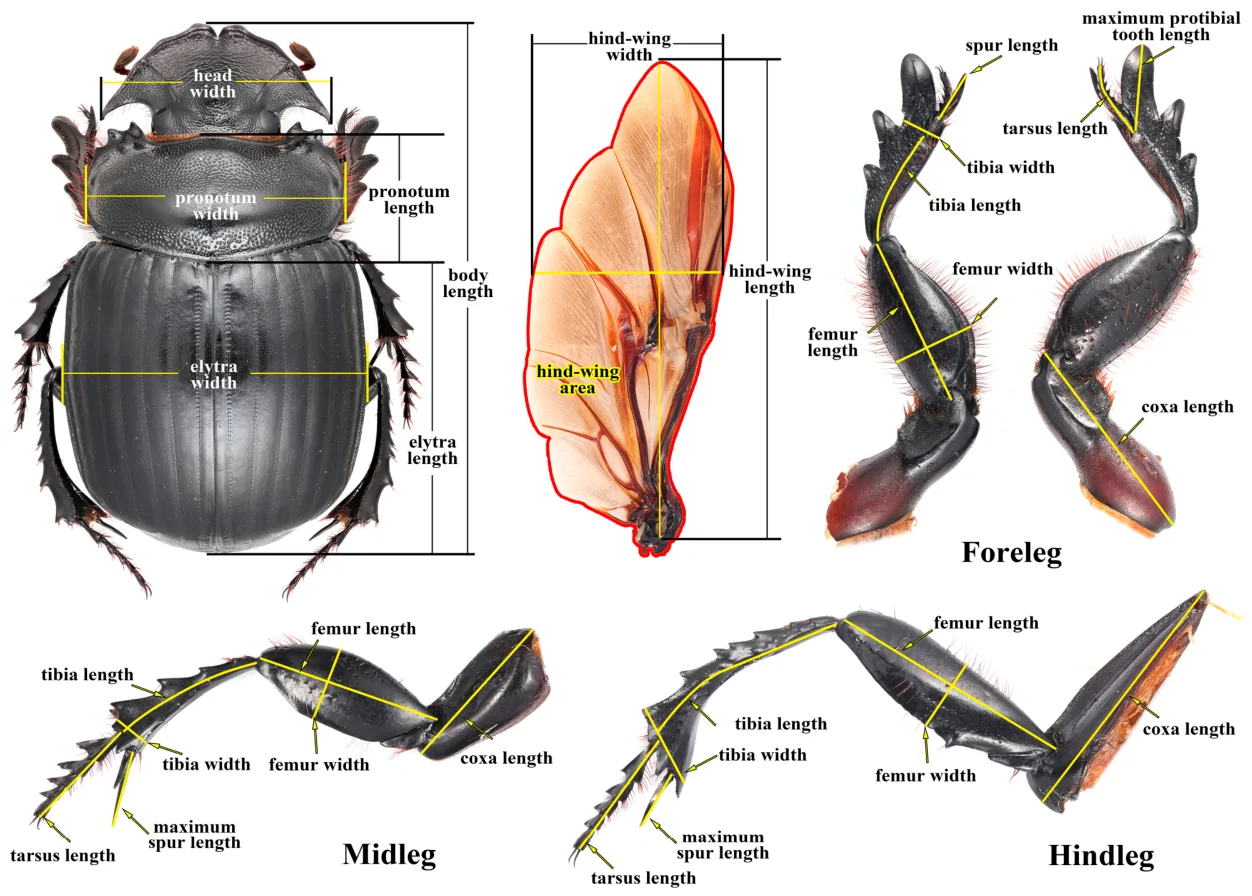
Demonstration of quantitative metrics for testing traits, using *Synapsis tridens* as an example. Yellow lines indicate the positions and orientations of the corresponding linear measurements.

Linear dimensions and hind-wing area were quantified from calibrated images using ImageJ v1.54. Measurements included body length, head width, pronotum length and width, elytral length and width, lengths of the coxa, femur, tibia and tarsus, femoral and tibial widths, number and maximum length of the external protibial teeth, lengths of the apical tibial spurs, and hind-wing length, width and area (Table 1). Measurement landmarks and definitions are illustrated in Figure 2.

Total fore, mid and hindleg lengths were calculated as the sums of femur, tibia and tarsus lengths. Coxa length was excluded from total leg length because the coxa primarily functions as the proximal articulation between the leg and thorax and differs mechanically from the serial distal segments contributing to appendage extension. Structures that could not be measured reliably were recorded as missing values and were not estimated or assigned a value of zero.

### 2.3. Functional Trait Derivation and Multivariate Analysis

To reduce the direct influence of overall body size and to quantify functionally relevant structural variation, proportional traits were derived from the raw measurements. Relative leg lengths were calculated by dividing total leg length by body length. Relative protibial tooth length and relative fore-tibial spur length were calculated as maximum tooth length and spur length, respectively, divided by protibial length. Relative hind-wing area was calculated as hind-wing area divided by squared body length, whereas hind-wing aspect ratio was calculated as squared hind-wing length divided by hind-wing area.

To reduce redundancy among correlated structural variables and avoid over-interpretation of trait-specific patterns, eight representative traits were selected for focused univariate assessment. These traits captured major aspects of functional morphology related to excavation, locomotion, and body form, including fore-femur width-to-length ratio, fore-tibia width-to-length ratio, relative protibial tooth length, relative fore-tibial spur length, pronotum width-to-length ratio, relative midleg length, relative hindleg length, and hind-tibia length-to-width ratio. Width-to-length ratios were used for the fore-femur and fore-tibia to represent structural robustness, whereas the hind-tibia was expressed as a length-to-width ratio to represent elongation. Trait distributions were visualized using boxplots with individual observations superimposed. Species were arranged according to the predefined large- and small-bodied tunneler–roller contrasts, with *S. tridens* shown separately as the mixed-strategy species.

Principal component analysis (PCA) was used to characterize multivariate structural differentiation among species. Prior to PCA, pairwise Pearson correlations among candidate functional traits were examined to reduce redundancy. For highly correlated trait pairs ($\left|r\right|\geq 0.80$), one variable was removed based on redundancy and functional interpretability. Hind-tibia length-to-width ratio was excluded from the PCA because it was strongly correlated with both relative hindleg length and relative hind-wing area.

The final PCA included nine traits: fore-femur width-to-length ratio, fore-tibia width-to-length ratio, relative protibial tooth length, relative fore-tibial spur length, relative midleg length, relative hindleg length, relative hind-wing area, hind-wing aspect ratio and pronotum width-to-length ratio. All variables were centered and standardized to unit variance before analysis. Individual scores and species centroids were plotted in PC1–PC2 space, and loading vectors were used to interpret the major axes of structural variation. For graphical clarity, eight representative loading vectors were displayed in the final ordination plot, although all nine traits were included in the PCA.

### 2.4. Allometric and Size-Matched Comparisons

Body length and each response variable were log_10_-transformed. Because the absolute body-size ranges of the five species showed limited overlap, body size was centered within species to separate within-species size variation from large interspecific differences in mean body size. Specifically, centered body size was calculated as the log_10_-transformed body length of each individual minus the mean log_10_ body length of its species.

For each response trait, two linear models were fitted. The common-slope model included within-species-centered body size, species and sex as explanatory variables:

$${log}_{10}\left(trait\right)\sim {body\;size}_{within}+species+sex$$

A second model additionally included the interaction between within-species body size and species:

$${log}_{10}\left(trait\right)\sim {body\;size}_{within}\times species+sex$$

The two models were compared using nested-model F tests to determine whether allometric slopes differed among species. *p* values from the four species × body-size interaction tests were adjusted using the Benjamini–Hochberg false discovery rate (FDR) procedure. When the interaction was not supported, the common within-species slope was retained. The estimated common slope was subsequently compared with the theoretical expectation under isometry, with an expected slope of 1 for linear dimensions and 2 for hind-wing area. *p* values from the four tests of isometry were independently adjusted using the Benjamini–Hochberg procedure.

To evaluate whether structural differences associated with resource-use strategy showed consistent directions across body-size classes, two predefined size-matched species contrasts were examined: *S. typhon* versus *C. molossus* for the large-bodied contrast and *G. mopsus* versus *O. lenzii* for the small-bodied contrast. Effect direction was consistently defined as roller relative to tunneler.

For each trait and contrast, the log response ratio was calculated as follows, where $\bar{X}$ represents the group mean:

$$\ln RR=\ln\left(\frac{{\bar{X}}_{roller}}{{\bar{X}}_{tunneler}}\right)$$

Positive lnRR values therefore indicated larger trait values in rollers, whereas negative values indicated larger values in tunnelers. Uncertainty around each effect size was quantified using 10,000 nonparametric bootstrap resamples, with individuals resampled independently within the roller and tunneler groups. Percentile-based 95% confidence intervals were obtained from the bootstrap distributions, and effect sizes and confidence intervals were summarized using forest plots.

All statistical analyses were conducted in R (version 4.5.2). Data manipulation and visualization were performed primarily using the dplyr, purrr, ggplot2 and ggrepel packages. The emmeans package was used where estimated marginal trends were required, and linear-hypothesis tests associated with the allometric analyses were conducted using the car package. PCA was performed on centered and standardized variables using the prcomp function.

### 2.5. Terminology and Abbreviations

Terminology and abbreviations for the structural features used in this study were based on Snodgrass (1935) and Chapman et al. (2013) [18,19].

## 3. Results

### 3.1. Comparative Morphology of Functional Structures

Body size differed among the five selected species (Figure S1, Table S2). Mean body length ranged from 10.09 mm in *O*. *lenzii* to 38.64 mm in *S*. *tridens*. The two large-bodied species, *C*. *molossus* and *S*. *typhon*, showed similar body lengths, whereas *O. lenzii* (tunneler) and *G*. *mopsus* (roller) represented the smaller-bodied comparison. *S*. *tridens* exhibited the largest mean body length among the five species.

Structural traits associated with different functional structures showed distinct patterns among species (Table S3). Foreleg-related traits varied considerably among species but did not correspond consistently with tunneling or rolling strategies. Robustness-related traits, including fore-femur and fore-tibia width-to-length ratios, were relatively high in *C. molossus*, whereas similar patterns were not observed in the small-bodied-species comparison.

Posterior-leg proportions showed a stronger association with resource-relocation strategies. Roller species had relatively longer hindlegs and more elongated hind-tibiae than their size-matched tunneler counterparts. This pattern occurred in both the large-bodied comparison between *S. typhon* and *C. molossus* and the small-bodied comparison between *G. mopsus* and *O. lenzii*.

Wing-related traits differed among species in both relative area and shape. However, variation in hind-wing morphology did not show a consistent separation between tunneling and rolling strategies, suggesting that these traits may reflect additional factors related to flight and dispersal.

The observed variation involved multiple structural features, with posterior-leg traits showing the clearest association with rolling behavior.

### 3.2. Univariate and Multivariate Variation in Functional Morphology

#### 3.2.1. Variation in Individual Functional Traits

**Figure 3:**
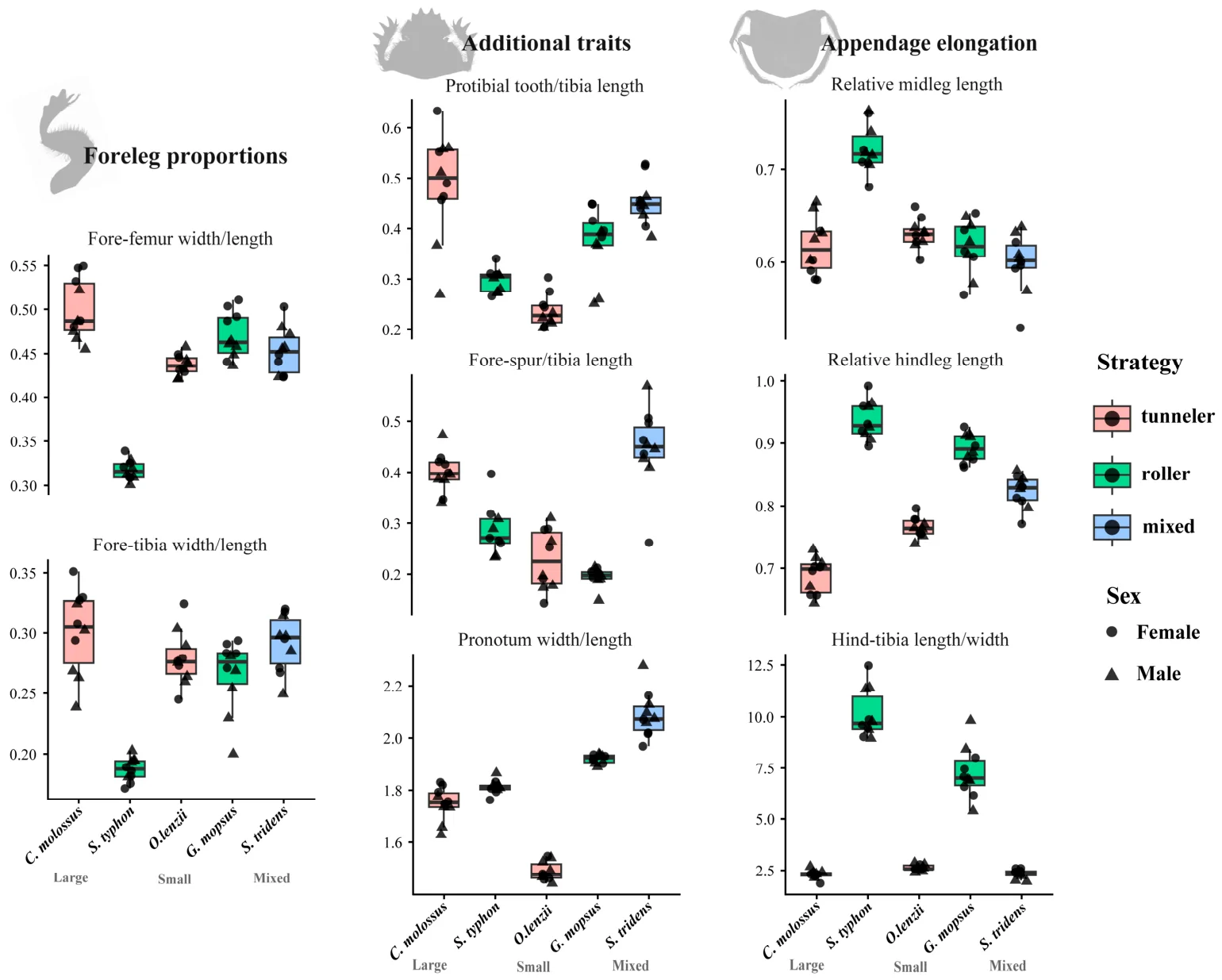
Variation in eight functional structural traits among five true dung beetle species with contrasting resource-use strategies. Boxes indicate the interquartile range (IQR), horizontal lines within boxes represent medians, and whiskers extend to values within 1.5 × IQR. Points represent individual specimens. Species are distinguished by color [and sexes by point shape, if applicable]. Fore-femur and fore-tibia width/length ratios represent structural robustness, whereas hind-tibia length/width represents elongation.

Clear interspecific variation was observed across the eight focal functional traits (Figure 3, Table S3). The patterns differed among structural features.

Foreleg-related traits showed substantial variation among species but did not exhibit a consistent separation between tunneler and roller species. The fore-femur width-to-length ratio was highest in *C*. *molossus* (0.500 ± 0.034), followed by *G*. *mopsus* (0.470 ± 0.026), whereas lower values were observed in *S*. *typhon* (0.319 ± 0.012) and *O*. *lenzii* (0.437 ± 0.012). A similar pattern was observed for the fore-tibia width-to-length ratio, with *C. molossus* showing the highest value (0.301 ± 0.035), while *S. typhon* showed the lowest value (0.187 ± 0.010). Relative protibial tooth length and relative apical spur length also varied among species, with higher values recorded in *C. molossus* and *S. tridens*.

Posterior-leg proportions showed a relatively consistent pattern with rolling behavior. Relative hindleg length was higher in roller species than in their size-matched tunneler counterparts, with *S. typhon* showing a higher value than *C. molossus* (0.937 ± 0.031 vs. 0.688 ± 0.029), and *G. mopsus* exceeding *O. lenzii* (0.892 ± 0.023 vs. 0.765 ± 0.016). A similar pattern was observed for hind-tibia length-to-width ratio, with markedly higher values in rollers (*S. typhon*: 10.1 ± 1.19; *G. mopsus*: 7.27 ± 1.25) than in tunnelers (*C. molossus*: 2.30 ± 0.208; *O. lenzii*: 2.62 ± 0.153). Relative midleg length showed weaker differentiation among these species.

Pronotum width-to-length ratio varied considerably among species, but did not show a consistent association with tunneling or rolling strategies. The mixed-strategy species *S. tridens* did not consistently occupy intermediate positions across the examined traits. Instead, it exhibited a combination of trait values, showing relatively high values in some traits (e.g., pronotum width-to-length ratio and protibial tooth/tibia ratio) but lower values in others (e.g., hind-tibia length-to-width ratio), indicating a mosaic structural configuration rather than an intermediate phenotype.

#### 3.2.2. Functional Morphospace

**Figure 4:**
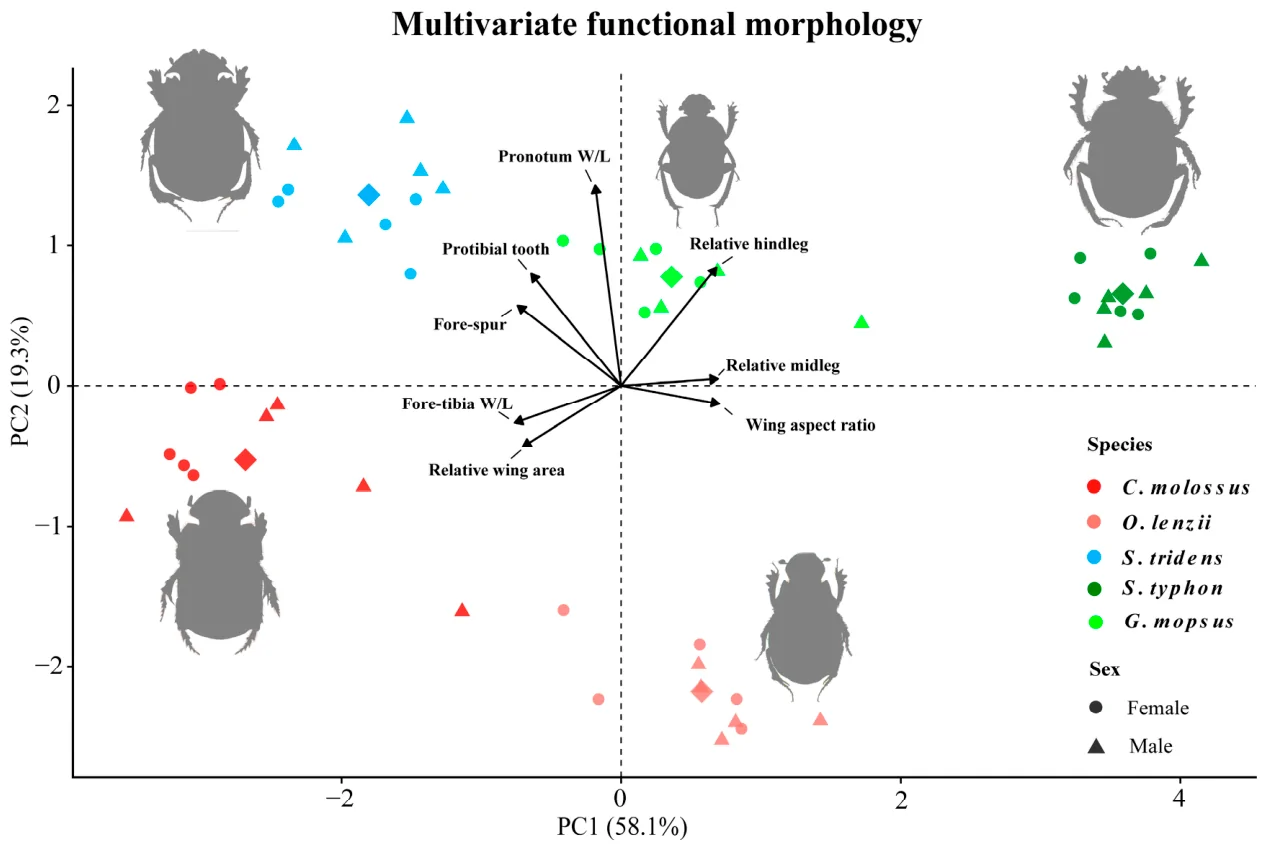
Functional morphospace of five dung beetle species based on principal component analysis of nine structural traits. PC1 and PC2 explained 58.09% and 19.26% of the total variation, respectively. Points represent individual specimens, and diamond symbols represent species centroids. Colors indicate species, and point shapes indicate sex. Arrows show the loadings of structural traits on the first two principal components.

The first two principal components explained 77.35% of the total robustness variation, with PC1 and PC2 accounting for 58.09% and 19.26%, respectively (Figure 4). PC1 mainly represented variation in appendage proportions and associated body-size-related structural differences. Negative PC1 scores were associated with relatively robust foreleg traits and higher relative hind-wing area, whereas positive scores were associated with relatively elongated mid and hindleg traits and higher hind-wing aspect ratio. PC2 was mainly associated with pronotum width-to-length ratio, with additional contributions from relative hindleg length and relative protibial tooth length.

Species occupied distinct positions within the functional morphospace. The large-bodied tunneler *C*. *molossus* showed the lowest PC1 score (−2.689 ± 0.725), whereas the large-bodied roller *S*. *typhon* showed the highest PC1 score (3.591 ± 0.270), representing the strongest separation among the predefined size-matched contrasts. The small-bodied roller *G*. *mopsus* showed a positive PC1 score (0.362 ± 0.609), whereas the small-bodied tunneler *O*. *lenzii* was positioned closer to the center of PC1 (0.577 ± 0.524), resulting in weaker separation between this size-matched pair. The mixed-strategy species *S*. *tridens* occupied a distinct region characterized by negative PC1 (−1.805 ± 0.444) and positive PC2 scores (1.360 ± 0.319), rather than an intermediate position between tunneler and roller species. The separation of species along PC1 and PC2 reflected contrasting combinations of body-size-related and structural variation, with different morphological modules contributing unequally to the observed differentiation.

### 3.3. Allometric Scaling and Size-Matched Functional Contrasts

#### 3.3.1. Allometric Relationships of Key Structural Traits

**Figure 5:**
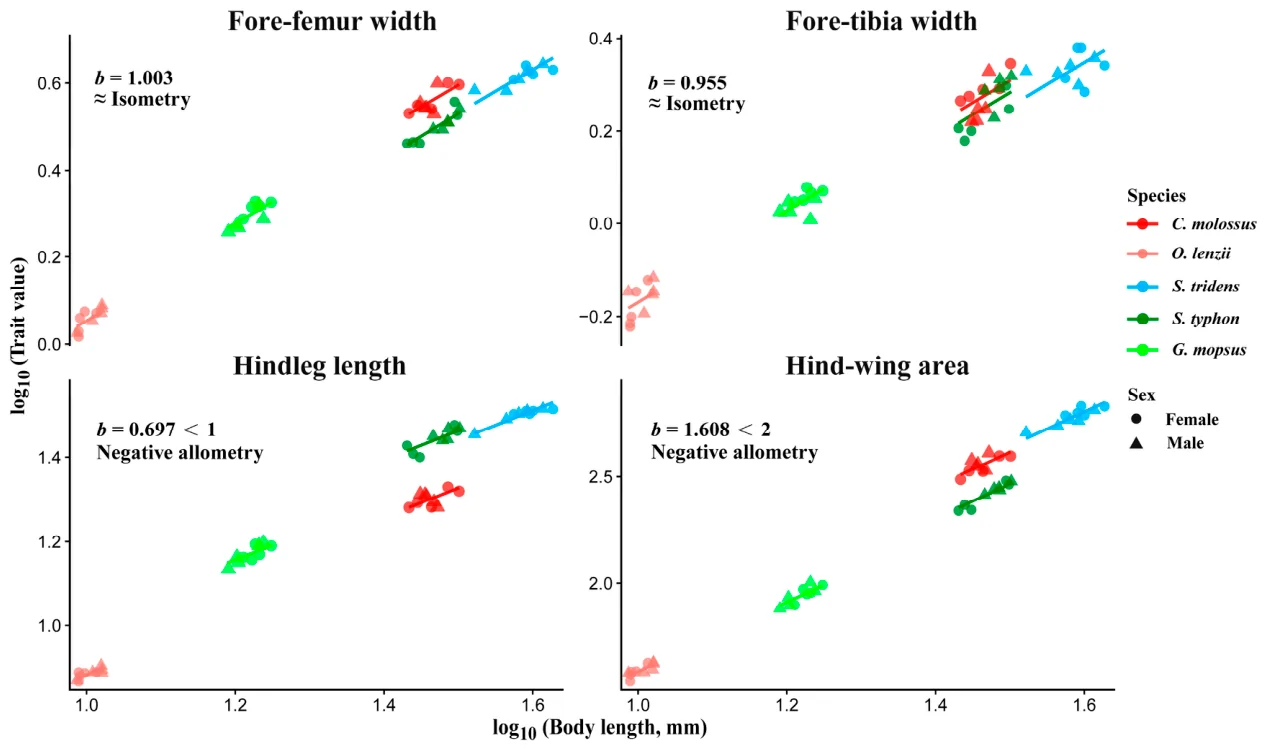
Allometric relationships between body size and structural morphological traits in five dung beetle species. Log_10_-transformed trait values were plotted against log_10_ body length. Each point represents an individual specimen; circles and triangles indicate females and males, respectively. Lines show fitted relationships for each species for visualization. Statistical inference was based on common-slope models including within-species body size, species, and sex. The slope coefficient (*b*) indicates the scaling relationship, with expected isometric values of 1 for linear dimensions and 2 for wing area. Deviations from these values indicate negative or positive allometry.

Species-specific allometric slopes did not differ significantly for any of the four structural traits after FDR correction (species × body-size interaction: FDR-adjusted *p* = 0.113–0.295; Table 2; Figure 5). Common within-species slopes were therefore retained for subsequent interpretation. Fore-femur width (*b* = 1.000, 95% CI = 0.779–1.230) and fore-tibia width (*b* = 0.955, 95% CI = 0.510–1.400) did not differ from the isometric expectation of *b* = 1 (both FDR-adjusted *p* = 0.982). In contrast, hindleg length showed significant negative allometry (*b* = 0.697, 95% CI = 0.531–0.862; FDR-adjusted *p* = 0.00249), indicating that hindleg length increased proportionally more slowly than body length. Hind-wing area was also negatively allometric relative to the area-based isometric expectation of *b* = 2 (*b* = 1.610, 95% CI = 1.310–1.910; FDR-adjusted *p* = 0.0236). Thus, within species, foreleg widths scaled approximately proportionally with body size, whereas hindleg length and hind-wing area increased at lower rates than expected under isometry.

Given the absence of significant among-species heterogeneity in allometric slopes, we next used size-matched species contrasts to assess whether structural differences between rollers and tunnelers were directionally consistent across body-size classes.

#### 3.3.2. Size-Matched Effect-Size Comparisons

**Figure 6:**
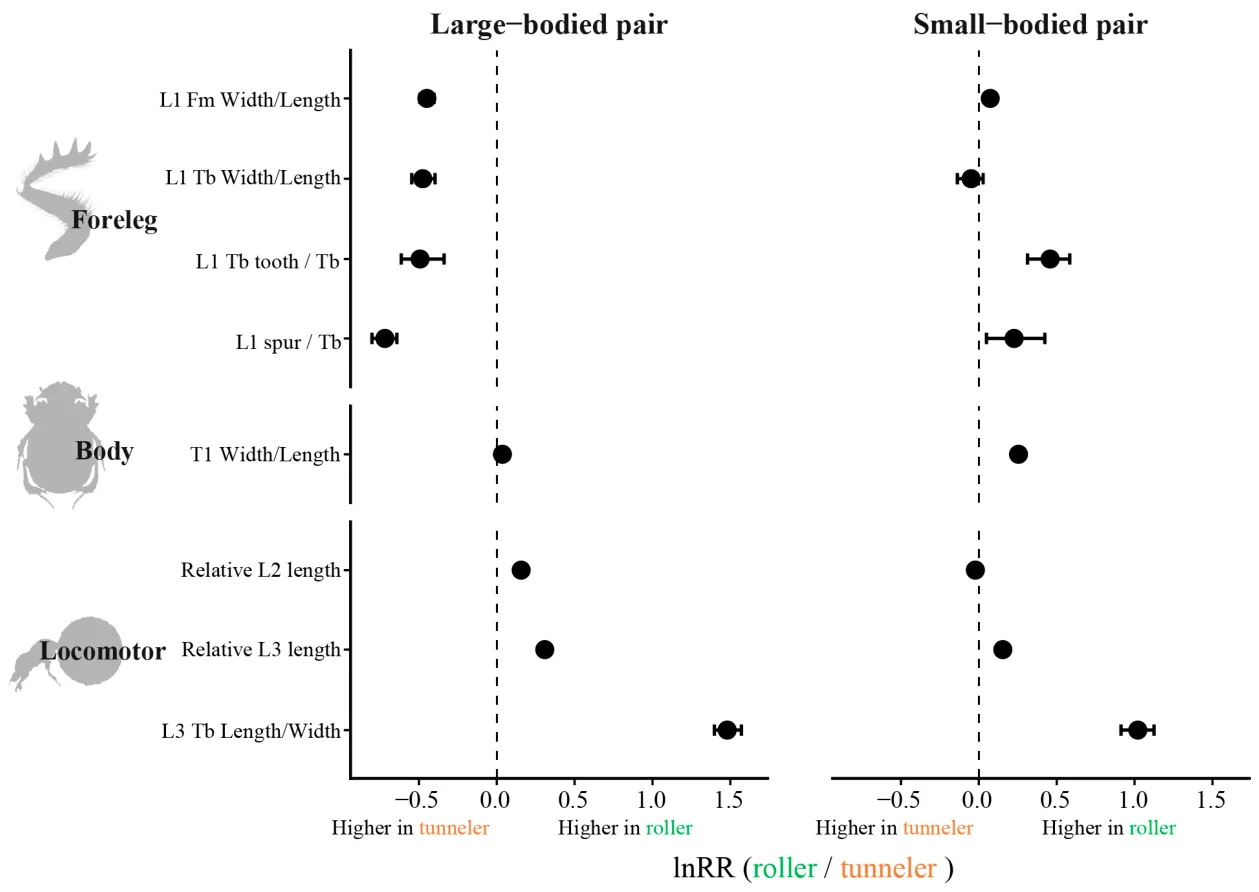
Size-matched contrasts in structural traits between roller and tunneler dung beetles. Effect sizes are expressed as log response ratios [lnRR = ln (mean roller/mean tunneler)] with 95% bootstrap confidence intervals based on 10,000 resamples. Black points represent lnRR estimates, horizontal black lines indicate 95% bootstrap confidence intervals, and the vertical dashed line denotes lnRR = 0. Positive values indicate larger trait values in rollers, whereas negative values indicate larger values in tunnelers. Large-bodied comparisons were made between *Scarabaeus typhon* and *Catharsius molossus*, and small-bodied comparisons between *Gymnopleurus mopsus* and *Onthophagus lenzii*.

Size-matched comparisons revealed that hindleg morphology showed the most consistent directional differentiation between resource-use strategies (Figure 6). Relative hindleg length was greater in rollers in both the large-bodied (lnRR = 0.308, 95% CI = 0.278–0.341) and small-bodied contrasts (lnRR = 0.154, 95% CI = 0.134–0.173), corresponding to approximately 36% and 17% greater values, respectively. Hind-tibia length-to-width ratio showed an even stronger and consistently positive response, with lnRR values of 1.48 (95% CI = 1.40–1.57) in the large-bodied contrast and 1.02 (95% CI = 0.913–1.13) in the small-bodied contrast. These values corresponded to approximately 4.4- and 2.8-fold greater hind-tibial elongation in the respective roller species.

Foreleg-related traits showed a less uniform pattern across the two body-size classes. Fore-femur and fore-tibia width-to-length ratios were lower in the large-bodied roller than in the corresponding tunneler. In the small-bodied pair, however, the fore-femur ratio was slightly higher in the roller, while the fore-tibia ratio differed little between the two species. Relative protibial tooth length and fore-tibial spur length also varied in direction between the large- and small-bodied contrasts. The pronotal width-to-length ratio was higher in the roller in both pairs, although the magnitude of the difference varied markedly between body-size classes.

These size-matched comparisons therefore point to hindleg elongation as the clearest recurring structural feature associated with rolling across the two body-size classes. Foreleg robustness and armature, by comparison, varied more strongly with species and body-size context. The allometric analyses characterized how individual traits scaled with body size, while the paired comparisons showed which structural differences were repeated between tunnelers and rollers at both body-size scales.

## 4. Discussion

### 4.1. Resource-Relocation Strategy and Locomotor Differentiation

Dung beetles exhibit remarkable diversity in dung-relocation behaviors, including vertical relocation by tunneling and horizontal relocation by rolling, which impose different mechanical demands on locomotion and substrate interaction [4,5]. In this study, the strongest and most consistent morphological pattern associated with resource-use strategy was observed in posterior-leg proportions. Both roller species showed higher relative hindleg length and greater hind-tibia length-to-width ratios than their size-matched tunneler counterparts, and this pattern was maintained across both large- and small-bodied comparisons.

The repeated differentiation of posterior-leg traits is consistent with previous observations that elongated posterior appendages facilitate dung-ball manipulation and rolling performance. Rolling dung balls requires coordinated movements of multiple leg pairs, with the mid and hindlegs contributing to ball manipulation, and stabilization during transport [20]. Structural comparisons across dung beetle assemblages have shown that leg proportions are associated with ecological roles and resource relocation strategies [10,11]. Recent biomechanical analyses further demonstrated that rolling species possess distinctive leg designs related to dung transport, particularly involving elongated hind-tibiae and modifications of leg functional architecture [21]. These findings, together with the present results, suggest that posterior-leg morphology represents a relatively reliable functional correlate of rolling behavior.

Foreleg traits showed a different pattern. Although *Catharsius molossus* exhibited relatively high fore-femur and fore-tibia width-to-length ratios, similar differentiation was not consistently observed in the small-bodied comparison. Protibial tooth and apical spur proportions also varied among species but did not show a repeated tunneler-associated pattern. Forelegs are involved in multiple functions, including soil excavation, substrate manipulation, and movement stabilization, and therefore may be influenced by several selective pressures rather than a single resource-relocation strategy. The absence of a simple tunneler–roller pattern in foreleg traits highlights that functional morphology should be interpreted through suites of correlated traits rather than isolated characters [14,15].

### 4.2. Body Size, Allometry, and Repeatability

Body size represents a major source of variation in insect morphology because many structures scale with overall body dimensions. Consequently, comparisons among species without accounting for size effects may confound ecological differentiation with allometric variation [21]. In the present study, size-matched comparisons between large-bodied and small-bodied tunneler–roller pairs were combined with allometric analyses to evaluate whether structural patterns were associated with resource-use strategy beyond simple body-size differences.

The consistent differentiation of relative hindleg length and hind-tibia proportions across size classes suggests that these traits are not merely consequences of larger body size in rollers. Instead, they represent repeated associations between locomotor morphology and dung-relocation behavior. Similar conclusions have been suggested in previous ecomorphological studies, where trait combinations rather than absolute body measurements provided better predictions of ecological function [10,13].

However, not all traits showed comparable repeatability. Scaling relationships may differ among structural modules because each structure experiences different functional constraints and evolutionary histories [17,21]. The mixed-strategy species showed partial overlap with the large-bodied tunneler in some allometric relationships, suggesting that species combining different resource-relocation behaviors may share certain scaling patterns rather than exhibiting a simple intermediate morphology. These results further emphasize that strategy-associated structural differentiation should be evaluated across multiple traits while considering their size dependence.

### 4.3. Differentiation Among Structural Modules

The examined morphological modules did not contribute equally to species differentiation. Locomotor traits showed contrasting patterns among functional modules: posterior-leg proportions, particularly elongation-related traits, exhibited the clearest and most repeatable association with rolling behavior, whereas robustness-related foreleg traits showed stronger differentiation in the large-bodied tunneler comparison. Wing-related traits and body proportions displayed weaker or more complex patterns.

Hind-wing morphology contributed to multivariate differentiation among species, particularly through relative hind-wing area and aspect ratio, but these traits did not show a consistent roller–tunneler separation. Wing morphology in beetles is shaped by both functional requirements and evolutionary constraints, and variation among species may reflect differences in dispersal ability, habitat use, and phylogenetic history rather than a single ecological strategy [22,23,24]. Studies of beetle flight have further demonstrated that wing and elytral structures influence aerodynamic performance and flight mechanics [25]. Therefore, the wing variation detected here likely represents broader flight-related differentiation rather than a distinct structural trait signature of dung-relocation behavior.

The multivariate analysis further supported this interpretation. Species were separated in morphospace by combinations of foreleg, posterior-leg, and hind-wing traits rather than by a single axis of structural variation. PC1 appeared to reflect a combination of body-size-related variation and functional morphology, whereas PC2 showed a clearer separation associated with resource-relocation strategy, with rollers and the mixed-strategy species tending toward positive scores and tunnelers toward negative scores. Notably, the mixed-strategy species *S. tridens* did not occupy an intermediate position between tunnelers and rollers, but instead exhibited a distinct combination of trait values. This finding suggests that behavioral categories may not always correspond to linear gradients in structural traits, particularly in species that combine multiple resource-use behaviors.

### 4.4. Generality and Future Directions

The present study focused on quantitative traits directly related to locomotion, resource relocation, and flight. However, dung beetle ecological specialization involves additional functional dimensions, including resource detection and processing. Previous studies have shown that mandibular morphology is associated with feeding ecology and dietary adaptation in Scarabaeinae [26], while antennal sensilla contribute to the detection of dung-derived chemical cues [27,28]. These structures were not included in the present framework because their functional roles require different quantitative approaches.

Future studies integrating locomotor, feeding, and sensory traits may provide a more comprehensive understanding of how ecological strategies are reflected across structural systems. In particular, combining geometric morphometrics, biomechanical analyses, sensory morphology, and broader phylogenetic sampling would help distinguish repeated functional adaptations from lineage-specific constraints. A limitation of the present study is the limited taxonomic sampling, with only five representative species examined. Expanding comparisons to additional species representing different body-size classes and resource-relocation strategies will be necessary to assess the generality of the identified trait associations across Scarabaeinae.

## Figures and Tables

**Table 1:** Structural traits used in the statistical analyses.

Functional Trait	Calculation	Functional Interpretation
Fore-femur width/length	Fore-femur width/fore-femur length	Foreleg robustness
Fore-tibia width/length	Fore-tibia width/fore-tibia length	Foreleg robustness
Relative protibial tooth length	Maximum protibial tooth length/protibial length	Protibial armature
Relative fore-tibial spur length	Fore-tibial spur length/protibial length	Protibial armature
Pronotum width/length	Pronotum width/pronotum length	Pronotal shape
Relative midleg length	Midleg total length/body length	Relative leg length
Relative hindleg length	Hindleg total length/body length	Relative leg length
Hind-tibia length/width	Hind-tibia length/hind-tibia width	Hind-tibial elongation
Relative hind-wing area	Hind-wing area/body length^2^	Relative wing area
Hind-wing aspect ratio	Hind-wing length^2^/hind-wing area	Wing shape

**Table 2:** Summary of allometric analyses for four key structural traits in five dung beetle species.

Trait	FDR-Adjusted *p* for Interaction	Common Slope (*b*)	95% CI	Isometric Expectation	FDR-Adjusted *p* for Isometry	Scaling Pattern
Fore-femur width	0.113	1.000	0.779–1.230	1	0.982	Isometry
Fore-tibia width	0.113	0.955	0.510–1.400	1	0.982	Isometry
Hindleg length	0.295	0.697	0.531–0.862	1	0.00249	Negative allometry
Hind-wing area	0.295	1.610	1.310–1.910	2	0.0236	Negative allometry

*b*, common within-species allometric slope estimated from models using species-centered log_10_ body size. FDR-adjusted *p* values were obtained using the Benjamini–Hochberg procedure. Isometric expectations were *b* = 1 for linear traits and *b* = 2 for area.

## Data Availability

The data that support the findings of this study are available in the Supplementary Material for this article. Further inquiries can be directed to the corresponding authors.

## Supplementary Materials

The following supporting information can be downloaded at: https://www.mdpi.com/article/10.3390/insects17090968/s1, Figure S1: The forelegs, midlegs, hindlegs, and wings of five true dung beetles. Table S1: Terminology and abbreviations for the structural features used in this study; Table S2: Raw data of measurements and derived trait data; Table S3: PCA results. Code S1: The code that was used in R for the variation in eight structural traits among five true dung beetle species; Code S2: The code that was used in R for the functional morphospace of five dung beetle species based on principal component analysis of nine structural traits.
